# Transcatheter edge-to-edge repair for complex mitral regurgitation: a case report of prolapse with leaflet perforation

**DOI:** 10.1093/ehjcr/ytaf242

**Published:** 2025-05-15

**Authors:** Yumi Yamamoto, Yasuhide Mochizuki, Ryota Kosaki, Hiroto Fukuoka, Toshiro Shinke

**Affiliations:** Division of Cardiology, Department of Medicine, Showa University School of Medicine, 1-5-8 Hatanodai, Shinagawa-ku, Tokyo 142-8555, Japan; Division of Cardiology, Department of Medicine, Showa University School of Medicine, 1-5-8 Hatanodai, Shinagawa-ku, Tokyo 142-8555, Japan; Division of Cardiology, Department of Medicine, Showa University School of Medicine, 1-5-8 Hatanodai, Shinagawa-ku, Tokyo 142-8555, Japan; Division of Cardiology, Department of Medicine, Showa University School of Medicine, 1-5-8 Hatanodai, Shinagawa-ku, Tokyo 142-8555, Japan; Division of Cardiology, Department of Medicine, Showa University School of Medicine, 1-5-8 Hatanodai, Shinagawa-ku, Tokyo 142-8555, Japan

**Keywords:** Mitral valve prolapse, Perforation, Healed infective endocarditis, Mitral transcatheter edge-to-edge repair, Transoesophageal echocardiography, Case report

## Abstract

**Background:**

Transcatheter edge-to-edge repair (TEER) is an established treatment for high-risk surgical candidates with severe mitral regurgitation (MR). However, its application in cases with leaflet perforation is rarely reported.

**Case summary:**

An 86-year-old woman presented with worsening dyspnoea. Transthoracic echocardiography revealed severe primary MR with P2 prolapse. Transoesophageal echocardiography (TOE) demonstrated chordae tendineae rupture and a 2.8 mm wide perforation at P2, suggestive of healed infective endocarditis. The distance from the tip of P2 in front of the perforation and the far end was measured to be ∼5.0 and 7.0 mm, measured using 3D multi-planar reconstruction. Given the patient's high surgical risk, TEER was planned after careful heart team discussion. The procedure successfully achieved intended grasping on the first attempt using one MitraClip® (XTW), reducing MR to mild without leaflet injury. One-week follow-up echocardiography showed no leaflet injuries or single leaflet device attachment. The patient's heart failure symptoms improved, with no recurrence or infection for a year.

**Discussion:**

Although TEER is not primarily recommended for MR with a perforation even in a patient at high surgical risk, this case demonstrates that TEER can be a viable option for high surgical risk patients with mitral valve prolapse and perforation when guided by detailed pre-operative TOE evaluation and careful heart team decision-making. The proximity of perforation to leaflet edge and use of appropriate clip size were crucial for successful repair.

**Learning points**

- The primary treatment strategy for severe mitral regurgitation with perforation should prioritize surgical intervention.
- In well selected cases of mitral valve prolapse causing regurgitation, if the infective endocarditis has been adequately treated and healed, transcatheter edge-to-edge repair (TEER) is feasible.
- TEER can be achievable for a case with mitral leaflet perforation when detailed pre-operative assessment is conducted with 3D multi-planar reconstruction analysis derived from transoesophageal echocardiography.

## Introduction

Transcatheter edge-to-edge repair (TEER) is a beneficial option for high-risk surgical candidates with symptomatic heart failure (HF) and severe degenerative mitral regurgitation (MR).^1^ However, there are very few reported cases of transcatheter treatment for primary MR with leaflet injury, such as perforation. Herein, we report a case of severe primary MR with a perforation in the prolapsed mitral valve, which was successfully treated using TEER with MitraClip® system (Abbott, Santa Clara, CA, USA).

## Summary figure

**Figure:**
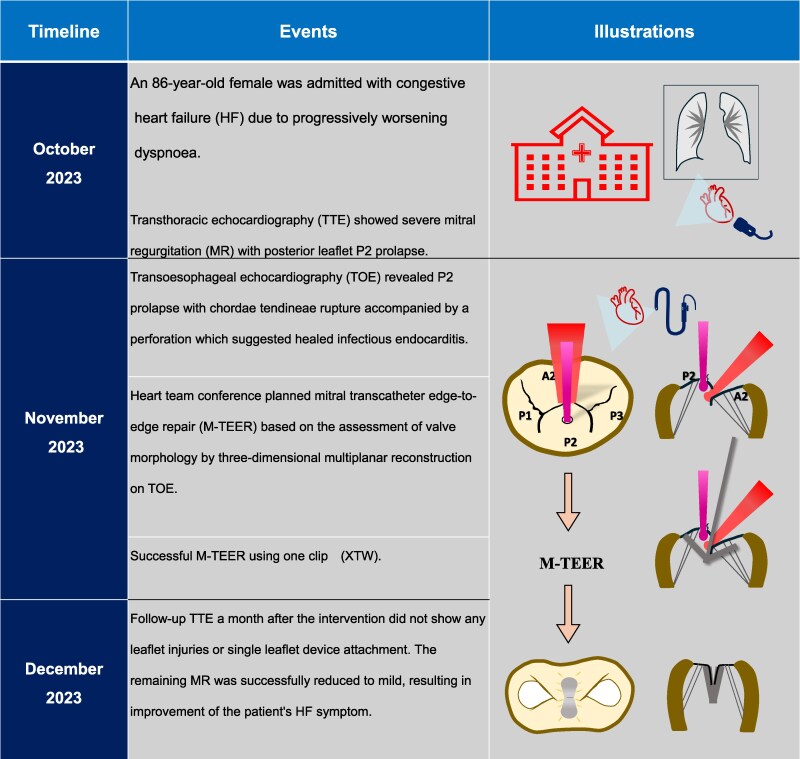


## Case presentation

**Figure 1:**
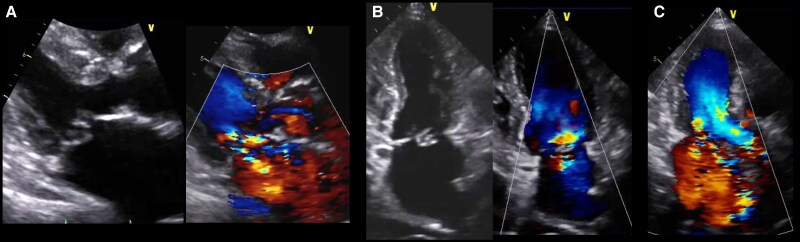
Severe mitral regurgitation with a posterior leaflet prolapse detected by transthoracic echocardiography on admission: (*A*) parasternal long axis view, (*B*) apical two-chamber view, and (*C*) apical three-chamber view.

**Figure 2:**
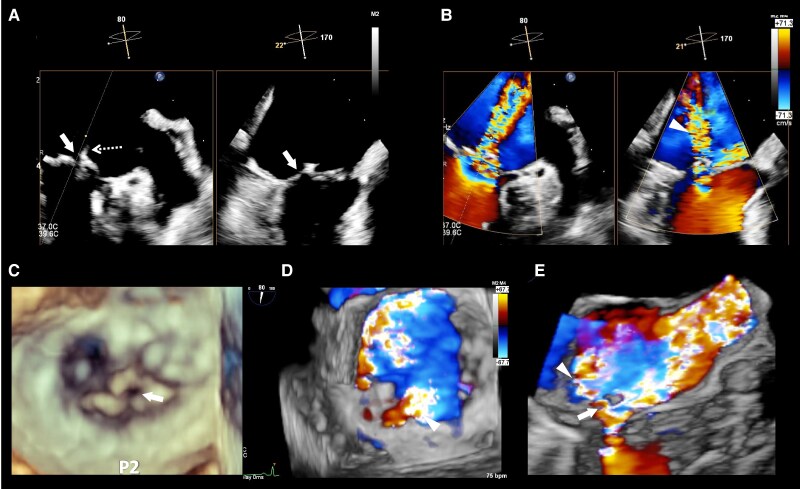
Pre-operative transoesophageal echocardiography (TOE). (*A*) Simultaneous visualization of two orthogonal planes showing the tissue discontinuity of posterior leaflet (arrow) and a string-like echo (braille arrow) on the left atrial side suggested a perforation of the posterior leaflet. In the same plane as (*A*), a jet directed posteriorly towards the left atrial roof (arrowhead) is observed at the perforation site on colour Doppler imaging (*B*). The P2 perforation determined in 3D TOE image (*C*). On 3D colour Doppler imaging, MR caused by P2 prolapse blowing anteriorly and a jet blowing posteriorly through the perforation were observed separately (*D*, *E*). Graphical elements; arrow = perforation of posterior leaflet, braille arrow = string-like echo on mitral valve nearby the perforation, arrowhead = MR jet towards the backward due to the perforation. MR, mitral regurgitation; P2, the middle segment of the posterior leaflet.

**Figure 3:**
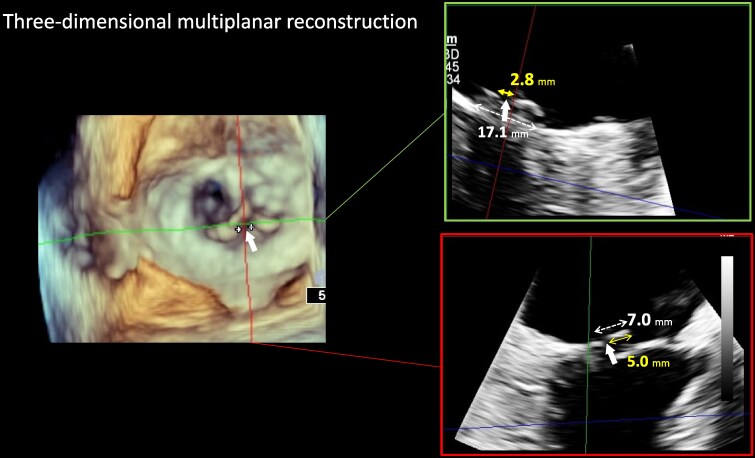
The location and size of the perforation measured by 3D multi-planar reconstruction (3d-MPR) method on transoesophageal echocardiography. The distance from the tip of P2 to the anterior edge of the perforation (white arrow) was 5.0 mm, and its posterior edge was 7.0 mm. The transverse diameter of the perforation was 2.8 mm, and the frailty width was 17.1 mm. P2: the middle segment of the posterior leaflet.

**Figure 4:**
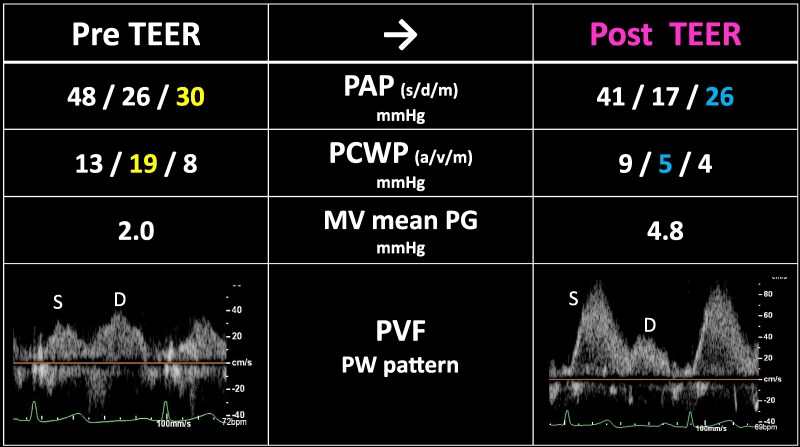
The changes of pressure parameters measured by right heart catheter and mean pressure gradient through mitral valve, and pulmonary vein flow pattern from transoesophageal echocardiography before and after transcatheter edge-to-edge repair (TEER). PAP, pulmonary artery pressure; PCWP, pulmonary capillary wedge pressure; MV, mitral valve; PG, pressure gradient; PVF, pulmonary vein flow; PW, pulse wave Doppler; S, systolic flow; D, diastolic flow.

An 86-year-old woman with New York Heart Association functional classification class III symptoms and progressively worsening dyspnoea was referred to our hospital for treatment of congestive HF. She also had a history of hypertension. The vital signs of the patient were as follows: Heart rate, 67 beats/min; blood pressure, 95/43 mmHg; and SpO_2_, 95% (room air). Physical examination revealed jugular vein distention, Levine-Ⅳ degree pan-systolic murmur, and mild leg oedema. The 12-lead electrocardiogram indicated normal sinus rhythm of 71 beats per minute. The chest X-ray suggested pulmonary congestion and bilateral pleural effusions. Blood examination revealed a slightly elevated B-type natriuretic peptide (BNP) level of 108 pg/mL (reference range: < 100 pg/mL) and renal dysfunction (creatinine clearance = 30 mL/min). The white blood cell count was 5500/mm³ (reference range: 4000–10 000/mm³), and the C-reactive protein level was 0.1 mg/dL (reference range: < 0.3 mg/dL). Transthoracic echocardiography (TTE) revealed end-diastolic/systolic left ventricular (LV) dimensions of 39/25 mm, LV ejection fraction of 67%, left atrial volume of 48 mL/m^2^. A qualitative assessment indicated severe primary MR with mitral valve prolapse (MVP) of the posterior leaflet. (*Figure 1*). This suggests the course of chronic MR, as indicated by left atrial enlargement and relatively low BNP levels. Transoesophageal echocardiography (TOE) revealed severe MR due to P2 prolapse with chordae tendineae rupture and most likely perforation at the centre of P2 (*Figure 2*; Supplementary material online, Movie  *S1*). A small strand was observed on the left atrial side of the mitral valve perforation, suggesting the presence of remnant torn tissue. Right heart catheter (RHC) revealed a cardiac index of 1.82 mL/min/m^2^, pulmonary artery pressure (PAP) of 52/19/31 (s/d/m) mmHg, and pulmonary capillary wedge pressure (PCWP) of 20/26/19 (a/v/m) mmHg. Furthermore, when the leg positive pressure (LPP) manoeuvre was applied, the PAP and PCWP increased to 59/20/35 (s/d/m) mmHg and 26/49/29 (a/v/m) mmHg, respectively. *Figure 3* shows the 3D multi-planar reconstruction (3D-MPR) method for TOE. The distance from the tip of P2 in front of the perforation and the far end was measured to be ∼5.0 and 7.0 mm. The transverse length of the perforation was measured to be 2.8 mm. In addition, the total length of P2, measured from base to tip, was 14 mm. Two sets of blood cultures yielded negative results. These findings strongly suggested non-active infective endocarditis (IE) with simultaneous evidence of severe MR from MVP and perforation due to past IE. At our heart team conference, this patient was judged inoperable with a high surgical risk due to age, frailty, and renal dysfunction. The predicted risk of mortality from EuroSCORE II was 15.9%. Based on the results of pre-analysis on TOE, transcatheter repair of the coaptation between A2 and P2 with simultaneous coverage of the perforation using the XTW (MitraClip®) was deemed feasible and planned, although tearing of the perforation due to the clip was of greatest concern. The procedure successfully achieved grasping at the intended position on the first attempt and was completed without leaflet injury, and the MR grade was reduced to mild (see Supplementary material online, Movie  *S2*). Technically, after lowering the gripper on the anterior leaflet, the Clip arm was slightly moved towards the posterior leaflet to maximize leaflet insertion at the P2 area. The changes in PAP and PCWP measured by RHC, mean pressure gradient through the mitral valve, and pulmonary vein flow pattern derived by TOE before and after TEER are shown in *Figure 4*. The PAP and PCWP decreased, and in the pulmonary venous waveform, the S wave became dominant over the D wave, indicating haemodynamic improvement. Follow-up TTE 6 months after the intervention did not reveal any leaflet injuries or single leaflet device attachment and residual MR was controlled to mild level (see Supplementary material online, Movie  *S3*). Mild degree of aortic regurgitation remained unchanged from the pre-operative assessment. Postoperatively, the patient showed an improvement in HF symptoms and no signs of HF recurrence or infection for one year.

## Discussion

This case could be considered valuable as the first report of a successful TEER for MVP with perforation, which is considered inactive IE. Active endocarditis is a definite contraindication for TEER^2^; however, in the case of healed IE, even with perforation, careful planning of TEER with TOE makes it possible to repair with the MitraClip®.

**Figure 5:**
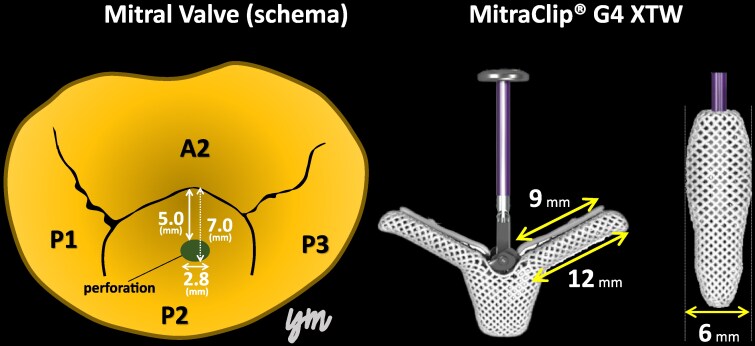
The relationship between a schema showing the surgeon's view of the mitral valve obtained from 3D multi-planar reconstruction on transoesophageal echocardiography and the actual MitraClip® G4 XTW.

Nishiura *et al*. reported that iatrogenic leaflet perforation caused by intraoperative clip manipulation could be treated with a second clip in a TEER case of functional MR.^3^ In this report, when the distance between the leaflet tip and perforation is within 10 mm, there is a possibility that coverage can be achieved by XTW or XT clips. In this case, the distance from the tip of P2 to the posterior edge of the perforation was 7.0 mm and the width of the perforation was 2.8 mm, thus the perforation was completely covered with the XTW because the XTW is designed with a 12 mm long, 6 mm wide arms and a 9 mm long grippers (*Figure 5*). In another case of an 80-year-old male with perforation in a cystically degenerated P2, the perforation was closed with a 10 mm AMPLATZER™ Muscular VSD Occluder after active IE was ruled out.^4^ Simultaneously, TEER was performed using XTW to repair the coaptation between A2 and P2. The detailed location of the perforation, such as its size and distance from the tip of the leaflet, is not indicated on TOE. In our case, pre-operative TOE with 3D-MPR analysis allowed mitral valve repair using a single XTW clip. Although the use of TEER for leaflet perforation remains debatable, it can sometimes be effective in high-risk surgical cases. A detailed pre-operative assessment of the number, diameter, and positional relationship of perforations within the valve using 3D-TOE analysis is essential. There have been several reported cases in which TEER has been performed for healed IE.^5–7^ However, a case involving a post-procedure leaflet tear necessitated surgical repair.^6^ Additionally, TEER carries the risk of causing post-procedural IE.^8^ Therefore, close monitoring using regular echocardiography and blood tests is essential during the postoperative period. It is recommended to minimize the grasping attempts as much as possible, since any further manipulation could lead to an increased risk in tearing the leaflet. Transcatheter mitral valve implantation remains a valuable therapeutically alternative, providing that it is available and applicable.^9^

## Conclusion

TEER may be considered an interventional option for high-risk surgical patients with degenerative MR accompanied by leaflet perforation. However, this case should by no means be considered a recommendation for TEER in MVP with perforation, as solid evidence is required regarding the chronic nature of the condition, and confirmation that the perforation is small, not fragile, and can be completely covered by the clip arms. The feasibility of TEER depends on a detailed pre-operative echocardiographic evaluation, and decision-making by an experienced heart team is crucial.

## Supplementary Material

ytaf242_Supplementary_Data

## Data Availability

The anonymized data underlying this article will be shared upon reasonable request with the corresponding authors.

## Lead author biography

Dr. Yumi Yamamoto graduated from Chiba University in 2020 and currently works in the Department of Cardiology at Showa University, specializing in cardiac imaging for structural heart disease.

## References

[1] Makkar RR, Chikwe J, Chakravarty T, Chen Q, O'Gara PT, Gillinov M, et al Transcatheter mitral valve repair for degenerative mitral regurgitation. JAMA 2023;329:1778–1788. 37219553 10.1001/jama.2023.7089 PMC10208157

[2] Hausleiter J, Stocker TJ, Adamo M, Karam N, Swaans MJ, Praz F. Mitral valve transcatheter edge-to-edge repair. EuroIntervention 2023;18:957–976. 36688459 10.4244/EIJ-D-22-00725 PMC9869401

[3] Nishiura N, Kubo S, Maruo T, Kadota K. Bailout clipping of a leaflet perforation during mitral transcatheter edge-to-edge repair using a larger clip size: a case report. Eur Heart J Case Rep 2023;7:ytad438. 37719004 10.1093/ehjcr/ytad438 PMC10500417

[4] Addis DR, Law M, von Mering G, Ahmed M. Codeployment of a percutaneous edge-to-edge mitral valve repair device and a ventriculoseptal defect occluder device to address complex mitral regurgitation with leaflet perforation. Catheter Cardiovasc Interv 2020;96:1333–1338. 32735734 10.1002/ccd.29147 PMC7680454

[5] Ninios V, Tourmousoglou C, Jancovici S, Kalin J. Percutaneous repair of healed endocarditis of the mitral valve using MitraClip devices around a large mobile vegetation. EuroIntervention 2019;14:1742–1743. 30398969 10.4244/EIJ-D-18-00973

[6] Kato Y, Amaki M, Kanzaki H, Kataoka Y, Okada A, Miyamoto K, et al MitraClip therapy for healed infective endocarditis-how long should we wait after active infection? Circ J 2019;84:130. 31597888 10.1253/circj.CJ-19-0523

[7] Chandrashekar P, Fender EA, Al-Hijji MA, Chandrasekaran K, Rihal CS, Eleid MF, et al Novel use of MitraClip for severe mitral regurgitation due to infective endocarditis. J Invasive Cardiol 2017;29:e21–e22. 28145876

[8] Frerker C, Kuck KH, Schmidt T, Kreidel F, Bader R, Schmoeckel M, et al Severe infective endocarditis after MitraClip implantation treated by cardiac surgery. EuroIntervention 2015;11:351–354. 25136886 10.4244/EIJY14M08_09

[9] Russo G, Gennari M, Gavazzoni M, Pedicino D, Pozzoli A, Taramasso M, et al Transcatheter mitral valve implantation: current Status and future perspectives. Circ Cardiovasc Interv 2021;14:e010628. 34407621 10.1161/CIRCINTERVENTIONS.121.010628

